# Aberrant network topology of cortical-subcortical circuits in schizophrenia and bipolar disorder: a graph theory study

**DOI:** 10.3389/fpsyt.2026.1919266

**Published:** 2026-08-05

**Authors:** Li Zhang, Jinping Guo, Wenli Wang, Shoucui Xia, Gong-Jun Ji, Yanghua Tian, Kai Wang

**Affiliations:** 1 Depatment of Psychiatry, Affiliated Psychological Hospital of Anhui Medical University, Hefei, Anhui, China; 2 Depatment of Psychiatry, Hefei Fourth People's Hospital, Hefei, Anhui, China; 3 School of Mental Health and Psychological Sciences, Anhui Medical University, Hefei, China; 4 Anhui Province Key Laboratory of Cognition and Neuropsychiatric Disorders, Hefei, China; 5 Department of Neurology, the First Affiliated Hospital of Anhui Medical University, Hefei, China; 6 Department of Neurology, The First Affiliated Hospital of Anhui Medical University, Hefei, China

**Keywords:** schizophrenia, bipolar disorder, resting state functional magnetic resonance, brain functional network, graph theory

## Abstract

**Background:**

Schizophrenia (SCH) or bipolar disorder (BD) patients exhibit a variety of abnormalities in brain network organization and function. More comprehensive characterization of both shared and disease-specific network features could provide essential clues to the underlying pathogenesis of these disorders, as well as potential therapeutic targets.

**Objective:**

In this study, we used graph theory methods to examine the common and specific characteristics of brain network between schizophrenia and bipolar disorder.

**Method:**

The patients of schizophrenia (n = 89) , patients of bipolar disorder (n = 57) and healthy control (HC) subjects (n = 45) were recruited. Resting-state functional magnetic resonance images were analyzed with a group-level connectivity matrix (GCM) threshold varying from 0.10 to 0.40 in 0.02 steps.

**Results:**

Network synchronization at the global level was significantly reduced in both SCH and BD groups compared to the HC group. Patients also exhibited impaired nodal parameters (nodal degree and betweenness centrality), especially SCH patients, in the hippocampus, temporal lobe, parietal lobe, and occipital lobe. The SCH group demonstrated sparser edges [fewer functional connectivity (FC) pathways] within somatosensory-motor Module I, denser edges (stronger FC) within limbic Module V, and reduced edge density between Module I and Module IV (including the default mode network), while both SCH and BD patients displayed denser edges between attention control Module III and Module V.

**Conclusions:**

These results revealed global-level network abnormalities in both SCH and BD, with more extensive nodal- and module-level abnormalities in SCH. These distinct topological characteristics could be useful biomarkers for differential diagnosis as well as treatment guidance and response evaluation.

## 1 Introduction

Schizophrenia (SCH) is a complex and debilitating psychiatric disorder characterized by delusions, hallucinations, disorganized thinking, cognitive impairments, and negative symptoms such as a flat affect (1). The disorder afflicts approximately 1% of the global population and places considerable burdens on patients, families, and society (2). In fact, SCH is amongst the leading causes of long-term disability worldwide (3). In the clinic, SCH is diagnosed primarily based on observation of symptoms as there are no widely accepted objective biomarkers specific to the disease (4). Schizophrenia symptoms also partially overlap with other neuropsychiatric and neurological disorders, particularly bipolar disorder (BD) (5), further complicating accurate diagnosis. Identifying the fundamental mechanisms underlying SCH and linking neural signatures to multidimensional clinical symptoms could therefore aid in diagnostic and treatment development (6).

Recent advances in functional magnetic resonance imaging (fMRI) techniques have revealed abnormalities in brain network organization stemming from changes in functional connectivity (FC) among brain regions, including the frontal lobe, parietal lobe, occipital lobe, thalamus, and superior temporal gyrus (7–9). Indeed, a growing body of evidence suggests that SCH is a disorder of functional dysconnectivity among regions critical for reasoning, emotional regulation, and other higher-order brain functions (10–12) rather than a dysfunction within discrete, localized brain regions. Thus, underlying network abnormalities may function as biomarkers for diagnosis, prognosis, treatment guidance, and ongoing treatment evaluation.

These abnormalities in network connectivity involve a wide range of regions, but appear particularly common or severe among the frontal lobe, temporal lobe, and insula (13, 14). In addition, previous studies of functional brain networks (FBNs) have reported that the degree centrality (DC), a measure of the average number of convergent inputs to a given network region (or network node), is markedly reduced in the bilateral putamen and increased in the left superior frontal gyrus of SCH patients (15).

While SCH and BD can be distinguished by unique clinical features or differences in shared symptom severity, there is often considerably overlap in symptom profile, familial pattern, risk factors (including risk genes), outcome measures, and treatment responses (16, 17). Recent research suggested that SCH and BD shared some common neurobiological features of brain network abnormalities with dopamine receptors/transporter (18). However other study represented schizophrenia displayed the more pronounced abnormalities of functional connectivity (FC) in both extent and severity than BD. In SCH unique alterations are observed in the brain regions involving the visual, cognitive control (19) and multiscale critical brain networks, suggesting severe impact of psychotic states on the brain’s complexity and organizational states (20). Furthermore, structural connectivity within the default mode network (DMN) exhibited opposing effects across the two disorders (21). The above may imply both common and distinct patterns of brain network connectivity associated with SCH and BD. Consequently, there is a need to explore disorder-specific pathophysiology in the brain for enhanced diagnostic accuracy and to provide clues to pathogenesis.

In this study, we acquired resting-state magnetic resonance imaging (rs-fMRI) data from SCH and BD patients as well as from healthy controls (HCs), and applied automated anatomical labeling (AAL-90) (22) and parcellation schemes to produce functional connectivity networks (matrices) for each participant. These networks were then analyzed to identify disease-specific and common alterations in topological organization. We hypothesized that SCH and BD patient groups would show distinct as well as common patterns of functional connectome disruption related to symptom severity. The abnormalities described here may aid in clinical diagnosis and treatment optimization, and provide a foundation for future studies on pathogenic mechanisms and novel treatment strategies for these two major psychiatric disorders.

## 2 Materials and methods

### 2.1 Participants

Eighty-nine patients with SCH and 57 with BD type I were recruited from the Affiliated Psychological Hospital of Anhui Medical University between Feb. 1, 2024 and Nov. 30, 2025. In addition, 45 healthy control (HC) participants were recruited from the local community through advertisements.

Schizophrenia and BD type I were diagnosed by certified psychiatrists based on Diagnostic and Statistical Manual of Mental Disorders, 5th Edition (DSM-5) criteria. Exclusion criteria were as follows: (1) younger than 18 or older than 50 years, (2) past or current neurological disorders, (3) history of severe head injury, (4) electroconvulsive therapy within 3 months of the study, and (5) contraindications to MRI scanning or severe claustrophobia. Psychiatrists also assessed severity of psychotic symptoms using the Positive and Negative Syndrome Scale (PANSS) (4) and other scales on the day of MRI scans.

The study protocol was approved by the Institutional Review Board of the Affiliated Psychological Hospital of Anhui Medical University (HFSY-IRB-YJ-KYXM-ZL(2024-014-001)). All participants or their legal guardians provided informed written consent after a full explanation of study aims and procedures.

### 2.2 MRI acquisition

All structural and functional magnetic resonance images were acquired using a General Electric 3-T scanner (Discovery GE750w) at Anhui Mental Health Center (AMHC). Participants were instructed to remain still with their eyes closed but without falling asleep during the scan. Earplugs and foam pads were used to reduce scanning noise and minimize head motion, respectively. Functional images consisting of 217 echo-planar imaging volumes were obtained with the following parameters: echo time, 30 ms; repetition time (FT), 2,400 ms; matrix size, 64 × 64; flip angle, 90°; field of view, slice thickness, 3mm; 192 × 192 mm^2^ and 46 continuous axial slices covering the whole brain. (one voxel = 3 × 3 × 3 mm^3^). In addition, high spatial resolution T1-weighted anatomic images were acquired with the following settings: voxel size = 1 × 1 × 1 mm^3^; TR=8.16 ms; number of slices = 188; flip angle = 12°; echo time = 3.18 ms; field of view = 256 × 256 mm^2^, and slice thickness = 1mm.

### 2.3 MRI preprocessing

*The rs-fMRI* images were preprocessed using the Data Processing Assistant for Resting-state Functional MR Imaging toolkit (DPARSF, http://rfmri.org/DPARSF) (23) and the procedures described in our previous study (24). The following processing steps were applied to each patient dataset: discarding the first 10 time-points of the fMRI series to achieve a steady state; slice-timing and head-motion correction; removing data with head-movement exceeded 2mm of translation or 2 degrees of rotation in any direction; coregistration to respective structural images; elimination of white matter signals, cerebrospinal fluid signals, and 24 Friston head-motion parameters by regression; spatial normalization based on unified segmentation of structural images; smoothing with a 4-mm isotropic Gaussian kernel.2.4 Functional connectivity matrix construction. Finally, temporal band-pass filtering (0.01-0.10Hz) was performed. Then we conducted a motion scrubbing to remove the time points with high motion (the framewise displacement > 0.5), as well as 1 time points prior to and 2 time points following each of these high motion time points.

### 2.4 Functional connectivity matrix construction

The time series of all brain regions of interest (nodes) as defined by the AAL-90 automated brain atlas were calculated for each participant using the GRETNA toolbox (http://www.nitrc.org/projects/gretna/). Pearson’s correlation coefficients were then calculated for each node pair, yielding individual whole-brain functional connectivity matrices (N × N, where N=90 nodes). Only positive correlations were reserved while negative correlations were set to zero. We defined network edges by applying a sparsity thresholding procedure ranging from 0.10 to 0.40 in steps of 0.02. Each correlation matrix was then transformed into a binary format according to the sparsity threshold, with all values greater than the threshold set to 1 and all others set to 0.

### 2.5 Network analysis

A variety of graph metrics from global to nodal were calculated to quantitatively describe network properties, including measures of functional integration, segregation, and centrality. The threshold value for each was varied from 0.1 to 0.40 in steps of 0.02. All graph metrics were calculated using GRETNA.

#### 2.5.1 Global graph metrics

Global efficiency (GE), characteristic path length (LP), assortativity, hierarchy and synchronization were calculated for all individuals as global metrics. Global efficiency was calculated as the average inverse shortest path length (25) and LP as the average of all shortest paths between each possible node pair in the network. The index of synchronization measures the likelihood of all nodes fluctuating with the identical waveform (26), assortativity measures the likelihood that a node is linked to other “similar” nodes (27), and hierarchy is used to identify the presence of a hierarchical organization in a network (28).

#### 2.5.2 Nodal graph metrics

Nodal degree (DEG) and betweenness centrality (BC), the two most common measures of centrality, were calculated as nodal measures. Nodal degree is the number of links (edges) connected to a given node while BC is defined as the fraction of all the shortest paths through a given node. Nodes with high BC values can be considered hub nodes that integrate information from various parts of the network (25). We calculated the Area Under the Curve (AUC) across the entire threshold range as the values for Nodal degree (DEG) and betweenness centrality (BC).

#### 2.5.3 Network modularity

**Figure 1:**
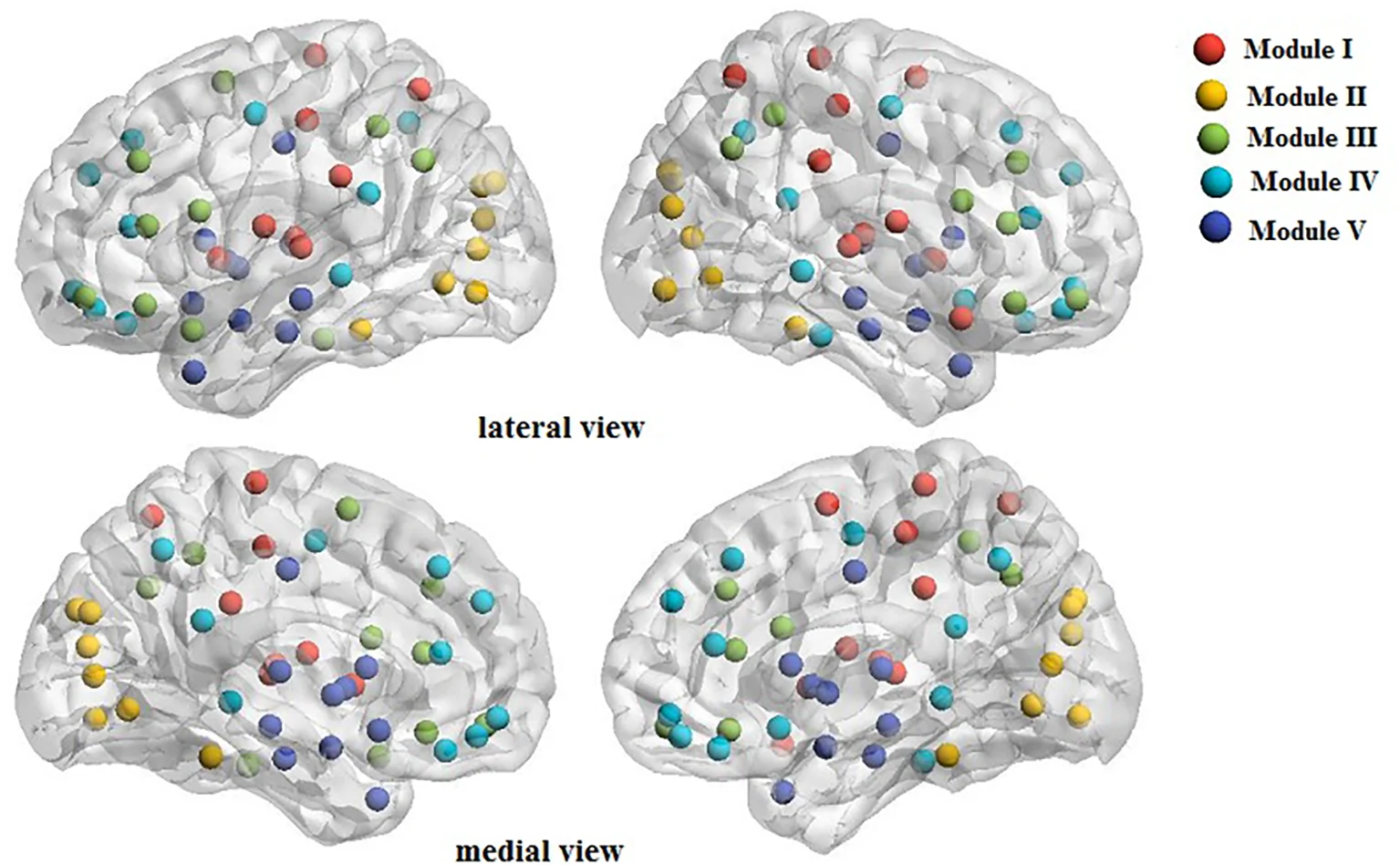
The modular structures of brain functional networks (constructed using BrainNet Viewer, http://www.nitrc.org/projects/bnv/) (29).

The complex whole-brain network was further partitioned into modules consisting of more strongly connected nodes. The partition coefficient (PC), intra-module functional connectivity (intra-FC), and inter-module FC (inter-FC) were calculated as module metrics for each individual subject, and then compared among groups. We defined five modules based on a previous article (29) (**Figure 1**): Module I responsible for somatosensory, motor, and auditory functions; Module II predominantly specialized for visual processing; Module III mainly involved in attentional processing by the frontal-parietal system;. Module IV including the default mode network (DMN); and Module V comprising limbic/paralimbic and subcortical systems involved in memory and emotion. Partition coefficients were calculated to assess the effects of individual nodes on the connections between modules (25). The sums of intra- and inter-modular edges (FC values above the given threshold) were then calculated for each participant. We defined the intra-module FC as the mean of total inter-nodal edges within the individual module, and inter-module FC as the mean of total edges between nodes in two different modules. The values for partition coefficients represent the area under the curve (AUC) across the entire threshold range.

### 2.6 Statistical analyses

Comparison of demographic variables

Continuous demographic variables were compared among groups by one-way analysis of covariance (ANCOVA) with *post hoc* two-sample t-tests for pairwise comparisons, while skewed variables were compared by the Mann-Whitney U-test. Sex ratio (the only dichotomous) demographic variable was compared among groups using the chi-squared test.

Comparison of network parameters

All network parameters were analyzed using MATLAB (Mathworks Inc., Natick, MA, USA). Global and regional network parameters were compared among groups by analysis of variance (ANOVA) with *post hoc* tests for pairwise comparisons. In addition, non-parametric permutation tests (10,000 repetitions each) (30, 31) were performed to detect group differences in nodal, global, inter-module, and intra-modular graph metrics. In each repetition, we randomly reassigned network measures and ANOVA F-values while maintaining the original subject numbers to obtain permutation distributions. Based on these distributions, p-values were measured for differences in network measures according to respective percentile positions, with significance set to 5% (the 95^th^ percentile). When indicated, we then conducted *post hoc* pairwise analyses at p<0.05 with false discovery rate (FDR) (32) correction for multiple comparisons. For all comparisons of network parameters (global, nodal, and module), sex, age, psychoactive medication dose, and education levels were regressed out as nuisance covariates.

Relationship between clinical variables and network parameters

Partial correlation analyses were conducted to assess associations between network parameters demonstrating significant group differences and clinical symptom severity as assessed by Positive and Negative Syndrome Scale (PANSS) scores, while controlling for sex, age, psychoactive medication dose, and education levels. A *p* <0.05 (FDR-corrected) was considered significant.

## 3 Results

### 3.1 Demographic and clinical characteristics of the study population

The demographic characteristics of the three groups are summarized in **Table 1**. There was no significant difference in sex ratio, mean age, and education level among groups. All patients were currently taking medication, and all antipsychotics prescribed were second generation. Medication load index was described in the previous article (33). No significant difference in medication load index was found between the two patients’ groups. As expected, PANSS scores were higher in the SCH group than the BD and HC groups (**Table 1**).

**Table 1:** Detailed demographics and clinical information of the study population.

Characteristic	SCH	BD	HC	Statistc	p value
Age, mean (SD)	35.6 (8.1)	32.9 (8.6)	34.9 (10.7)	F=1.522	0.221
Sample size (n)	89	57	45		
Male	28	19	16	χ2=0.231	0.891
Female sex, No	61	38	29		
education years, mean (SD)	11.1 (3.3)	11.9 (3.3)	12.1 (3.7)	F=1.365	0.258
duration of desease	11.1 (3.2)	10.2 (3.3)		t=0.804	0.423
Medication load index	2.35 (0.66)	2.40 (0.65)	NA	t=-0.394	0.694
Frame-wise Displacement, mean (SD)	0.04 (0.01)	0.04 (0.01)	0.03 (0.01)	F=1.212	0.3
Clinical characteristics					
PANSS_P, mean (SD)	15.1 (4.7)	11.6 (2.9)	7.8 (0.81)	F=42.9	<0.001
PANSS_N,mean (SD)	17.2 (5.8)	11.5 (2.8)	7.6 (0.7)	F=56.7	<0.001

SCH, schizophrenia; BD, bipolar disorder; HC, health control; SD, Standard. Deviation; PANSS, Positive and Negative Syndrome Scale; FD, the frame wise displacement.

### 3.2 Global parameters

**Figure 2:**
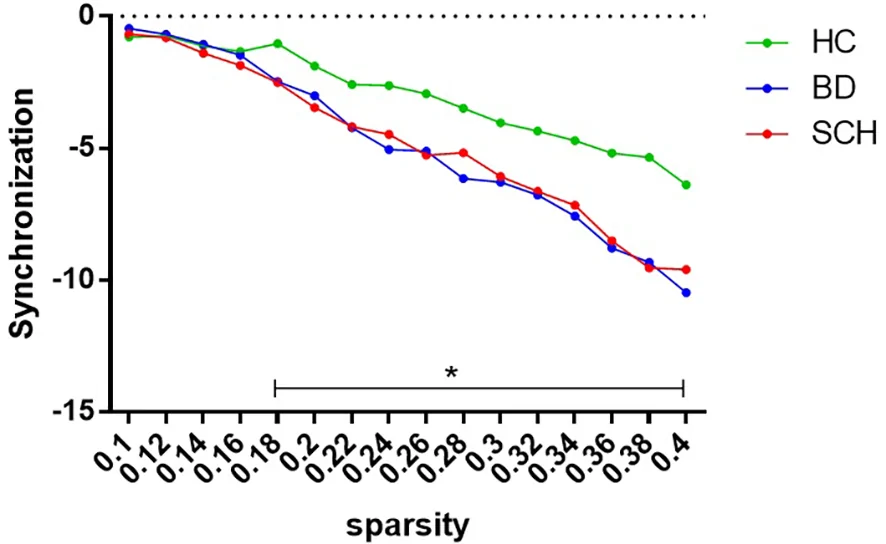
Comparison of global network parameters among schizophrenia (SCH) patients, bipolar disorder (BD) patients, and healthy controls (HCs). Network synchronization was lower in both SCH and BD patient groups compared to the HC group, while the other global network metrics examined (global efficiency, characteristic path length, and assortativity) did not differ among groups. Error bars represent standard errors. ^*^p < 0.05. The line above the horizontal axis represents the threshold range for significant differences among groups.

There were significant differences in synchronization (F=5.025, p = 0.007, η^2^=0.015) among the three groups, and *post hoc* analyses revealed significant reductions in both patient groups compared to the HC group (p < 0.05) (**Figure 2**). Alternatively, assortativity, global efficiency (GE), and hierarchy did not differ among groups (assortativity: F=0.641, p = 0.560; GE: F=2.400, p = 0.093; hierarchy: F=0.115, p = 0.892).

### 3.3 Nodal parameters

**Figure 3:**
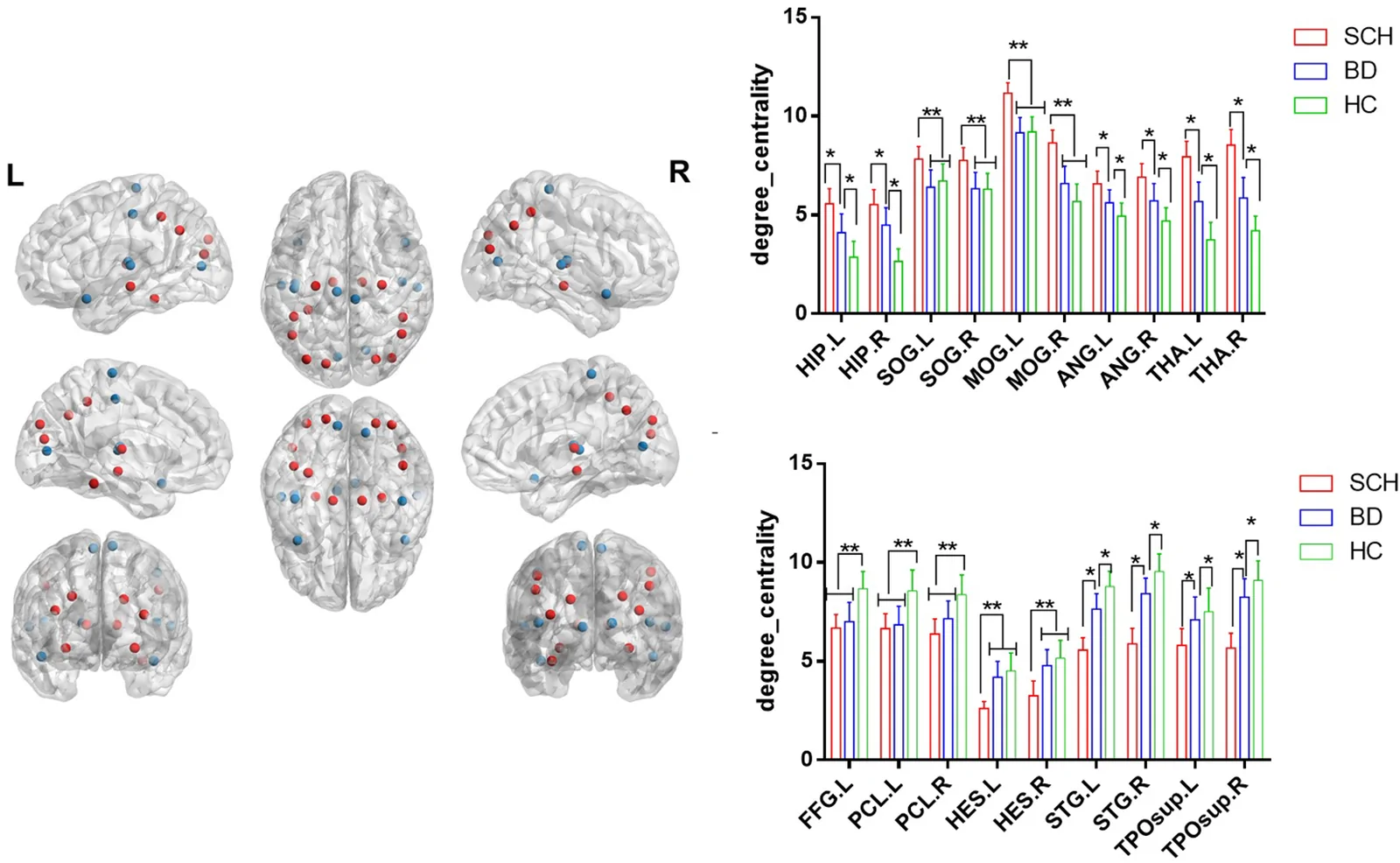
Comparison of the nodal degree parameter (DEG) among SCH; BD; and HC groups. Error bars represent standard errors. ^**^p < 0.01; ^*^p < 0.05. HIP_L, left hippocampus; HIP_R, right hippocampus; SOG_L, left superior occipital gyrus; SOG_R, right superior occipital gyrus; MOG_L, left middle occipital gyrus; MOG_R, right middle occipital gyrus; ANG_L, left angular gyrus; ANG_R, right angular gyrus; THA_L, left thalamus; THA_R, right thalamus; FFG_L, left fusiform gyrus; PCL_L, left paracentral lobule; PCL_R, right paracentral lobule; HES_L, left Heschl gyrus; HES_R, right Heschl gyrus; STG_L, left superior temporal gyrus; STG_R, right superior temporal gyrus; TPOsup_L, left temporal pole – superior temporal gyrus; TPOsup_R, right temporal pole – superior temporal gyrus.

Nodal degree (DEG) differed significantly among groups (**Figure 3**) in bilateral hippocampus (left: F=14.47, p < 0.001, η^2^=0.132; right: F=17.74, p < 0.001, η^2^=0.155), bilateral superior occipital gyrus (left: F=5.621, p = 0.004, η^2^=0.056; right: F=6.920, p = 0.001, η^2^=0.068), bilateral middle occipital gyrus (left: F=16.79, p < 0.001, η^2^=0.15; right: F=22.704, p < 0.001, η^2^=0.193), bilateral angular gyrus (left: F=8.408, p < 0.001, η^2^=0.081; right: F=11.397, p < 0.001, η^2^=0.107), and bilateral thalamus (left: F=31.97, p < 0.001, η^2^=0.252; right: F=35.23, p <0.001, η^2^=0.271), and *post-hoc* comparisons revealed highest values in the SCH group compared to the BD and HC groups (SCH > BD, SCH > HC). In addition, DEG values for the bilateral hippocampus, angular gyrus, and thalamus were higher in the BD group than the HC group (SCH > BD > HC). Nodal degree values also differed significantly among groups in the left fusiform gyrus (F=7.884, p = 0.001, η^2^=0.077), bilateral paracentral lobule (left: F=6.697, p = 0.002, η^2^=0.068; right: F=7.592, p = 0.001, η^2^=0.074), bilateral Heschl gyrus (left: F=11.884, p < 0.001, η^2^=0.111; right: F=8.465, p < 0.001, η^2^=0.082), bilateral superior temporal gyrus (left: F=29.244, p < 0.001, η^2^=0.235; right: p < 0.001), and bilateral temporal pole: superior temporal gyrus (left: p =0.019; right: F=28.023, p < 0.001, η^2^=0.228). In contrast to the bilateral hippocampus, in the superior occipital gyrus, middle occipital gyrus, angular gyrus, and bilateral thalamus, *post-hoc* comparisons revealed significantly lower values in the left fusiform gyrus, bilateral paracentral lobule, bilateral Heschl gyrus, bilateral superior temporal gyrus, and bilateral temporal pole. For the superior temporal gyrus of SCH patients compared to HCs, values in the left fusiform gyrus and bilateral paracentral lobule were lower in both patient groups compared to HCs.

**Figure 4:**
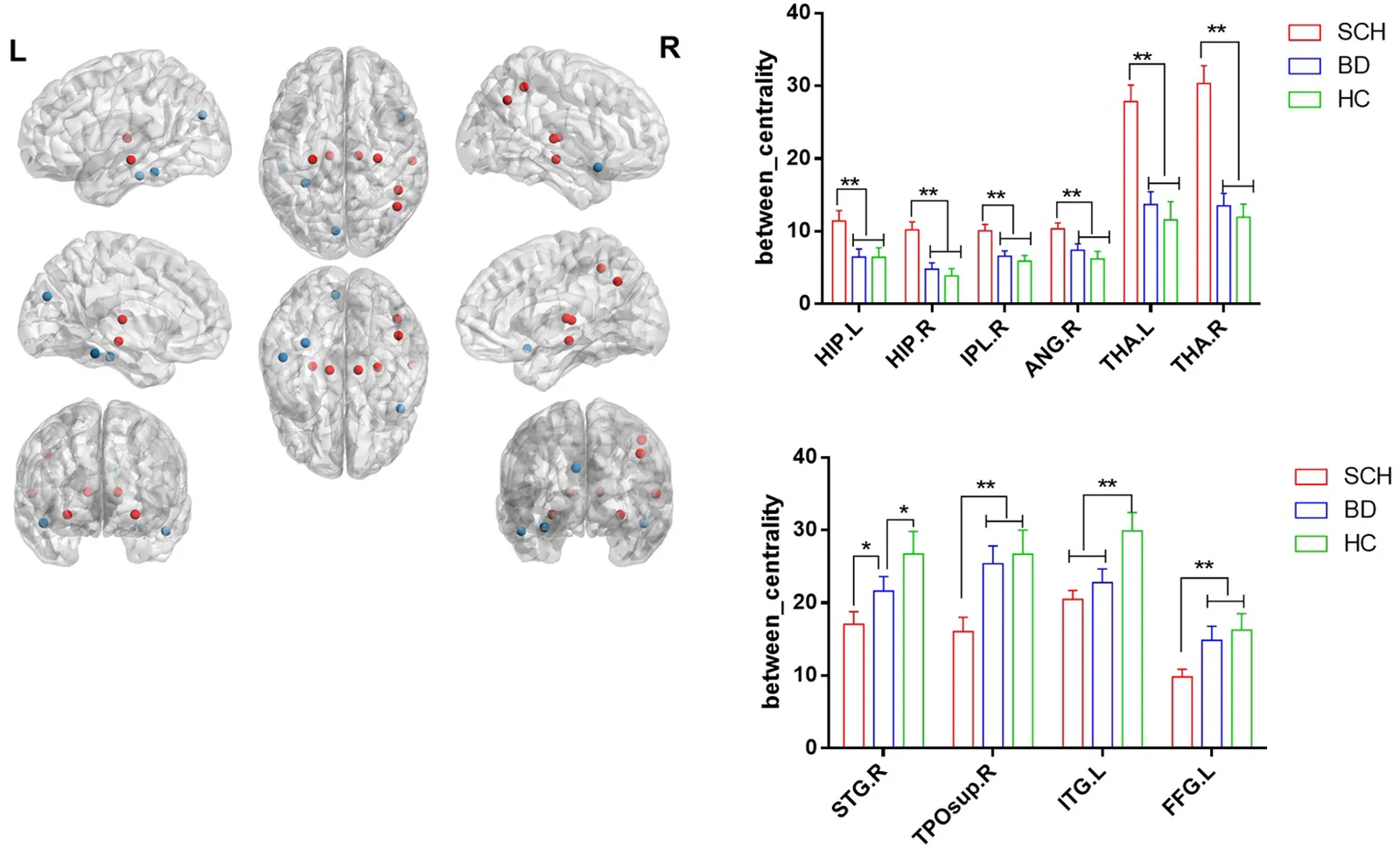
Comparison of nodal betweenness centrality (BC) among SCH, BD, and HC groups. Error bars represent standard errors. ^**^p < 0.01, ^*^p < 0.05. Abbreviations as defined in **Figure 3**.

Similarly, betweenness centrality values differed significantly among groups (all p < 0.05; **Figure 4**) and *post hoc* comparisons revealed significantly higher values among SCH patients compared to the other two groups in the bilateral hippocampus (left: F=4.831, p = 0.009, η^2^=0.048; right: F=8.425, p < 0.001, η^2^=0.081), inferior parietal, but supramarginal and angular gyri (F=7.250, p = 0.001, η^2^=0.071), right angular gyrus (F=6.846, p = 0.001, η^2^=0.067), and bilateral thalamus (left: F=20.053, p < 0.001, η^2^=0.174; right: F=23.834, p < 0.001, η^2^=0.201). Conversely, *post-hoc* testing revealed lower values among SCH patients compared to the HC group in the right superior temporal gyrus (F=6.226, p = 0.002, η^2^=0.062), right temporal pole: superior temporal gyrus (F=7.000, p = 0.001. η^2^=0.069), left inferior temporal gyrus (F=9.747, p < 0.001, η^2^=0.093), and left fusiform gyrus (F=5.401, p = 0.005, η^2^=0.054). *Post-hoc* comparisons also revealed lower values among the SCH group than the BD groups in the right temporal pole: superior temporal gyrus and left fusiform gyrus, and lower values in the left inferior temporal gyrus of both patient groups compared to the HC group. Finally, BC values followed the trend SCH< BD < HC in the right superior temporal gyrus (**Figure 4**).

### 3.4 Modularity

**Figure 5:**
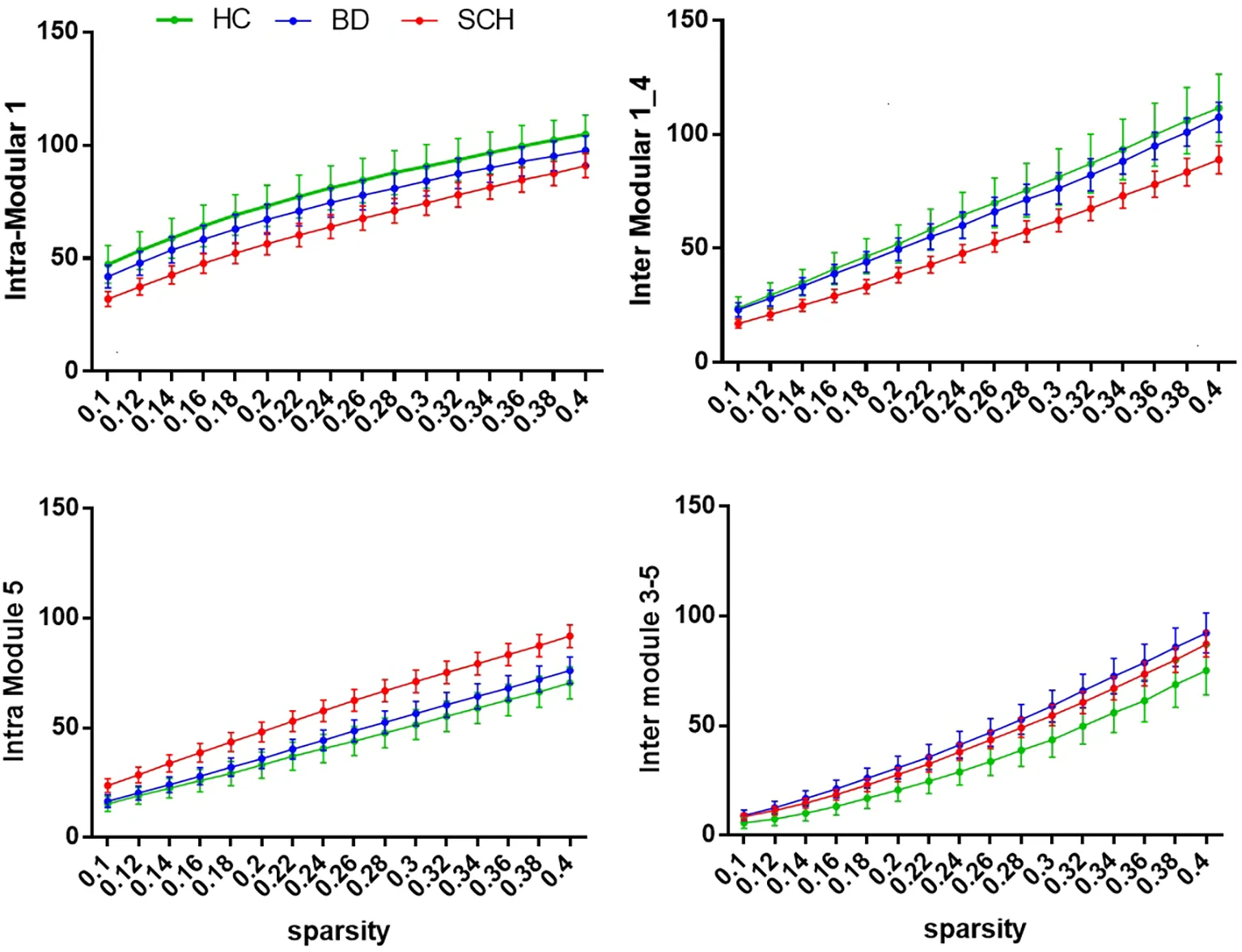
Comparison of edge density (functional connectivity pathway number) within Module I and Module V, between Module I and IV, and between Module III and V among SCH, BD, and HC groups. ^**^p < 0.01, ^*^p < 0.05. Module I includes somatosensory, motor and auditory cortex, Module V is comprised of limbic/paralimbic and subcortical networks, Module IV includes the default mode network, and Module III is the frontal-parietal network.

The SCH group exhibited sparser edges (fewer FC pathways above the given threshold) within Module I (p < 0.01) and denser edges (more FC pathways above threshold) within Module V (p < 0.01) compared to BD and HC groups. The number of edges between Module I and Module IV was also reduced in the SCH group, while the combined patient group exhibited denser edges between Module III and Module V compared to the HC group. (**Figure 5**).

**Figure 6:**
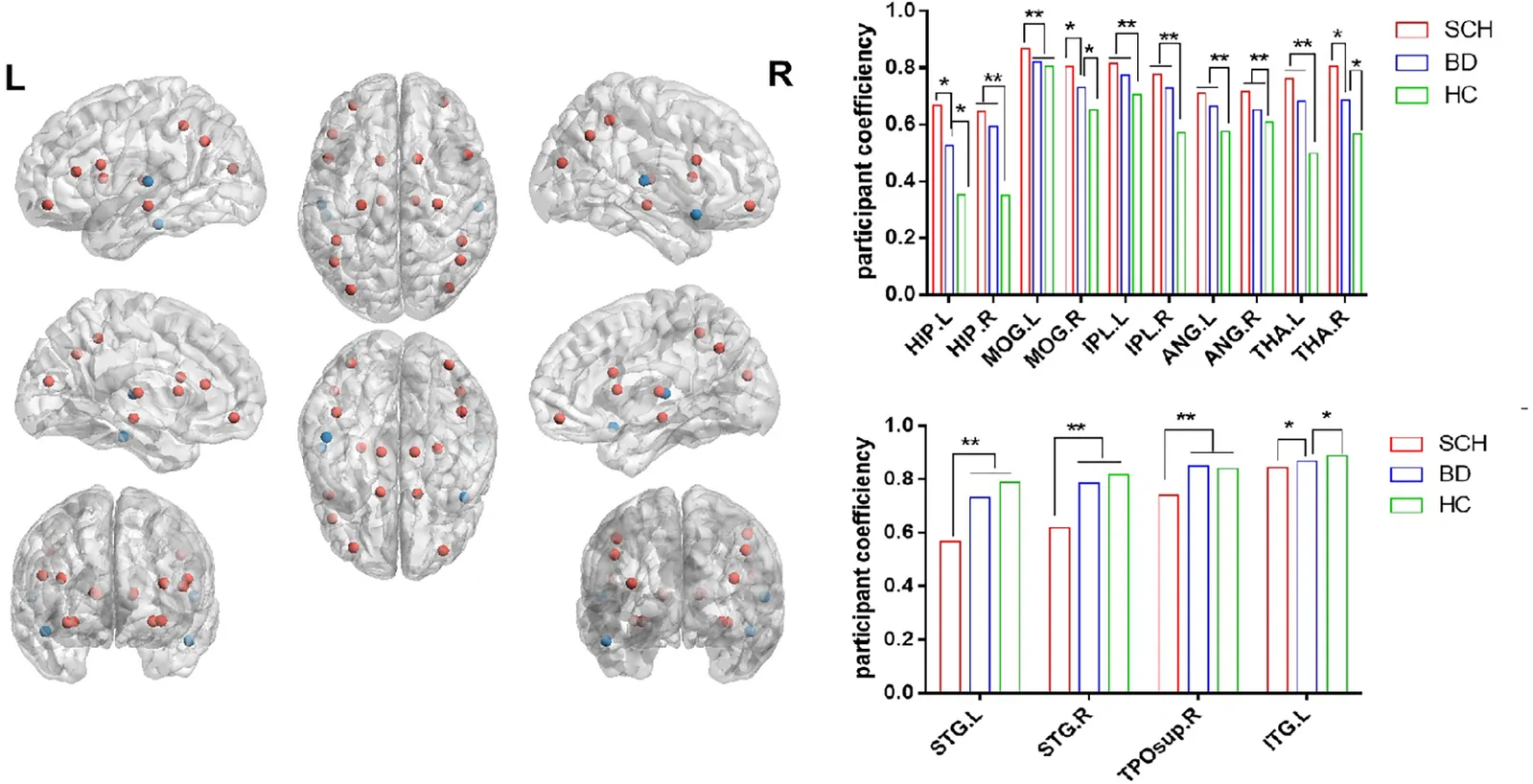
Comparison of the nodal partition coefficient among SCH, BD, and HC groups. Error bars represent standard errors. Abbreviations as defined in **Figure 3**.

Schizophrenia patients exhibited significantly greater partition coefficients (PCs) than BD and HC groups in the bilateral hippocampus (left, F=13.84, p < 0.001, η^2^=0.127; right, F=14.218, p < 0.001, η^2^=0.13), bilateral middle occipital gyrus (left, F=5.133, p = 0.007, η^2^=0.051; right, F=10.271, p < 0.001, η^2^=0.098), bilateral inferior parietal, but the supramarginal and angular gyri (left, F=8.389, p < 0.001, η^2^=0.081; right, F=16.211, p < 0.001,=0.146), bilateral angular gyrus (left, F=13.232, p < 0.001, η^2^=0.122; right, F=5.027, p = 0.007, η^2^=0.05), and bilateral thalamus (left, F=12.281, p < 0.001, η^2^=0.114; right, F=11.979, p < 0.001, η^2^=0.112) had different results. Alternatively, PC values were lower in the bilateral superior temporal gyrus (left, F=19.913, p < 0.001, η^2^=0.173; right, F=15.670, p < 0.001, η^2^=0.142), right temporal pole: superior temporal gyrus (F=7.432, p = 0.001, η^2^=0.073), and left inferior temporal gyrus (F=4.804, p = 0.009, η^2^=0.048) of SCH patients (**Figure 6**).

### 3.5 Relationship between network metrics and clinical variables

No significant correlation was detected between these network metrics and PANSS scores after FDR correction.

## 4 Discussion

Analyses of rs-fRMI data from SCH patients, BD patients, and matched HCs based on graph theory revealed both disorder-specific and shared abnormalities in neural network topology among patients. Our main findings were as follows: (1) at the global level, both SCH and BD groups showed significantly reduced network synchronization compared to HCs; (2) both patient groups also demonstrated nodal-level abnormalities in the hippocampus, temporal lobe, parietal lobe, and occipital lobe, which tended to be more severe in the SCH group; and (3) at the module level, the SCH group exhibited fewer edges (weaker FC) within (somatosensory-motor) Module I, denser edges (stronger FC) within (limbic/subcortical) Module V, and fewer edges between Module I and Module IV (including the default mode network, DMN), while both patient groups displayed denser edges between (frontoparietal) Module III and Module IV. Collectively, these findings revealed widespread network, subnetwork, and regional processing deficits in SCH and BD that may provide clues to pathogenesis and could provide neuroimaging biomarkers for differential diagnosis and treatment planning.

### 4.1 Weaker network synchronization in SCH and BD patients

Global network synchronization measures the likelihood of coincident activity among widespread nodes (i.e., fluctuating in phase). This network synchronization was reduced in both patient groups compared to the HC group, and more severely impaired in SCH than BD, consistent with previous studies reporting sensory integration deficits in both SCH and BD patient groups, but greater severity in the SCH group (34). Lower synchronization of activity across nodes suggested inefficient communication, resulting in poor integration of the disparate information required for complex neuropsychological functions such as cognition (35). Indeed, in both SCH and BD populations, diminished global neural communication was associated with impaired intellectual function, social cognition, and working memory (36) as well as emotional dysregulation and negative symptoms (37). The less severe synchronization deficit in BD may explain the milder clinical phenotype characterized by mood dysregulation but better preservation of most cognitive functions compared to SCH (38). These differences in network synchronization may thus provide clues to the neural substrates that differentiate psychiatric disorders with partially overlapping symptomatology.

### 4.2 Altered nodal parameters in BD and SCH

Patients with SCH or BD also exhibited abnormal nodal network metrics, mostly in the thalamus, hippocampus, temporal lobe, parietal lobe, middle occipital gyrus (MOG), and superior occipital lobe (SOG), and again these abnormalities appeared more severe in SCH than BD. These structures are crucial hubs implicated in both positive and negative symptoms, especially impaired motivation and goal-directed behavior (39). Emotional and motivational functions are modulated through the limbic system, and the current results suggested that the observed symptomatic differences between SCH and BD may be attributed to aberrant network architecture in limbic cortices. It has been suggested that the majority of brain regions are topologically isolated most of the time, with only a few connector hub regions, such as the hippocampus, serving as functional bridges. In other words, these nodes facilitate periods of global integration by linking otherwise functionally segregated structures (40). The hippocampus receives a disproportionately high level of information flow, thereby acting as a crucial convergence zone within the network (41). Therefore, disrupted topological structure within the hippocampus or between the hippocampus and various efferent and afferent structures could disrupt neural integration, potentially leading to SCH or BD symptoms. Moreover, the more severe this disruption, the more profound the dysfunction observed in these disorders.

The hippocampus is involved in learning, memory, visuospatial orientation, emotion regulation, and stress responses (42, 43), and so it is not surprising that hippocampal abnormalities are strongly implicated in both the psychotic and cognitive symptoms of SCH and BD (44, 45). Abnormalities in four neurotransmitter signaling pathways have been described in the hippocampus of both SCH and BD patients (46), which may be the neurochemical basis for overlapping symptomatology. These findings further suggested that the hippocampus is a core therapeutic target for both SCH and BD (47).

Both SCH and BD patients exhibited greater DEG in the SOG and MOG, possibly contributing to the impaired visual backward masking performance observed in these clinical groups, as both structures are of great importance for visual information processing (48). However, SCH is characterized by diffuse deficits impairing all stages of visual processing (49), while BD patients demonstrate disruptions only at the later stages involving higher-order attentional functions (50). Consistent with these findings, DG values in the SOG and MOG were higher among SCH patients than BD patients, indicating a greater deviation from the HC group. Thus, we speculate that altered visual information processing may represent a core dimension of BP and SCH, with greater severity in SCH.

The thalamus is a primary node controlling sensory input to cortical regions and cortical-cortical communication, so disruptions in thalamic network structure have broad deleterious effects on all levels of brain function from sensory perception to higher-order cognition (51). Structural defects have been found in thalamic association nuclei of both SCH and BD patient groups and linked to negative symptoms (49, 52), as well as disturbances of wakefulness, sleep, and memory (53).

Alternatively, we found reduced nodal values in the superior temporal gyrus (STG) and fusiform gyrus (FFG) only among SCH patients. Substantial abnormalities of the STG have been reported in SCH, which may contribute to both negative and positive symptoms. The STG contains the Heschl’s gyrus (HG) and planum temporale, which are major anatomical substrates for speech and language processing (54). The STG is also extensively connected to the limbic system, thalamus, and prefrontal cortex (55), so disrupted connectivity with the STG may contribute to auditory hallucinations, thought disorder, and cognitive deficits in SCH (56). Moreover, a longitudinal study demonstrated severe atrophy of the HG over time (57). In addition, structural abnormalities of the fusiform gyrus have been implicated in facial recognition deficits among SCH patients (58), while others have reported associations between fusiform gyrus volume and positive symptoms, including paranoid-hallucinatory syndrome (59). A recent study also reported that fusiform gyrus volume was reduced in SCH compared to major depressive disorder (MDD) and BD, and thus may be a useful biomarker for differential diagnosis (60).

However, no significant correlation was detected between these network metrics and PANSS scores after FDR correction, these above key regions showed altered degree and betweenness centrality in SCH (often SCH > BD > HC) may imply the abnormal topological structure partly related to the compensatory reorganization, or methodological artifacts related to thresholding, etc. Overall, these conclusions precluded evaluation of causality.

### 4.3 Similar and distinct modular-parameter changes in BD and SCH

Schizophrenia patients demonstrated multiple complex abnormalities in subnetworks defined by nodal FC strength, consistent with the multidimensional symptom profile of this disorder, including reduced edge number (weaker FC) within somatosensory-motor Module I, higher edge number (stronger FC) within limbic Module V, reduced edge number (weaker FC) between Module I and DMN Module IV, and higher edge number between frontoparietal Modules III and Module V. In contrast, only higher FC between Modules III and V was observed in BD.

Module I includes structures (nodes) responsible for somatosensory, motor, and auditory functions. Motor and somatosensory impairments have been consistently reported in antipsychotic-naive SCH patients, individuals at risk of developing the illness, and the biological relatives of patients, suggesting that such symptoms may arise from communication deficits (weaker FC) within Module I (61, 62). Furthermore, SCH patients have shown impaired somatosensory gating and lower pain sensitivity (63), slower motor responses, impaired involuntary movements, catatonia, and inappropriate tuning of motor and somatosensory functions (64–66), which again may stem from weaker FC in Module 1. Both the SCH and BD groups displayed weaker FC (fewer edges) between Module I and Module IV, which included the DMN involved in autobiographical memories and internal self-directed thought processes. The defective connection between Module I and Module IV (67) could therefore lead to abnormal DMN activity, which in turn may result in poor control of imagination, an inability to distinguish between imagination and reality, and ultimately to the development of psychotic symptoms such as hallucinations and bizarre ideation (68).

The SCH group also exhibited stronger FC within limbic/paralimbic and subcortical Module V and both disorder groups showed stronger FC between Module V and Module III encompassing the frontal-parietal attention system. Increased intra-module connectivity between limbic/paralimbic and subcortical systems may be related to clinical symptoms such as abnormal emotions and thoughts, while increased connectivity with Module III may result in excessive input to limbic/paralimbic and subcortical systems, biasing attention, leading to uncontrolled fluctuating emotions, and potentially also contributing to delusions.

While these network abnormalities may help explain the various symptoms of SCH and DB, the study had several limitations. First, the sample size was relatively small, so some important differences in network topology may have not reached statistical significance. Indeed, we found no significant correlation with clinical symptoms as measured by the PANSS. Second, the cross-sectional design precluded evaluation of causality. Future longitudinal observations are of particular interest to assess how these network abnormalities relate to symptom emergence and treatment response. Third, we did not distinguish among disorder subtypes such as first-episode versus chronic SCH. This heterogeneity could have further limited statistical power to detect specific network abnormalities and associations with symptoms. Fourth, relatively coarse brain template (AAL-90), which was selected to define the network nodes, reduced anatomical specificity and inter-subject variability compared to other subject-specific atlases (e.g. FreeSurfer). Fifth, we applied *a priori* based on prior literature to define network modules, the modular findings may partly reflect imposed assumptions rather than data-driven network organization. Finally, medication may have been a confounding factor, albeit an unavoidable one given the ethical considerations of keeping patients medication-free. Further studies using a prospective design and enrolling patients at first episode are needed to address these issues.

## 5 Conclusion

This study revealed both shared and disease-specific abnormalities of neural network topology in SCH and BD patients. These abnormalities in topological organization appeared more widespread and severe among SCH patients and so may help explain the more severe clinical phenotype compared to BD. Further studies with more homogenous patient populations (such as drug-naïve first-episode patients) are warranted to elucidate how these network abnormalities contribute to individual symptom profiles, and to identify promising network biomarkers and therapeutic targets.

## Data Availability

The original contributions presented in the study are included in the article/supplementary material. Further inquiries can be directed to the corresponding author.
